# Elevated atherogenic index of plasma is associated with increased cardiorenal syndrome prevalence: a cross-sectional study

**DOI:** 10.1080/0886022X.2025.2472037

**Published:** 2025-03-02

**Authors:** Sikai Xu, Jianping Hu, Zhiyi Ouyang, Maolin Yuan, Yan Zheng, Xin Liu, Yang Shen

**Affiliations:** a Department of Medical Genetics, the Second Affiliated Hospital of Nanchang University, Nanchang, China; b Jiangxi Key Laboratory of Molecular Medicine, The Second Affiliated Hospital of Nanchang University, Nanchang, China; c Huan Kui College of Nanchang University, Nanchang, China; d Department of Nephrology, The Second Affiliated Hospital of Nanchang University, Nanchang, China

**Keywords:** Cardiorenal syndrome, Atherogenic Index of Plasma, triglycerides, high-density lipoprotein cholesterol, dyslipidemia

## Abstract

**Purpose:**

Cardiorenal syndrome (CRS) is a complex clinical condition characterized by the simultaneous dysfunction of the heart and kidneys. The atherogenic index of plasma (AIP), calculated as the logarithm of the ratio of triglycerides (TG) to high-density lipoprotein cholesterol (HDL-C), has emerged as a potential biomarker for cardiovascular risk. This study investigates the association between AIP and CRS, aiming to explore the potential linkage between AIP and CRS.

**Methods:**

Data were sourced from the National Health and Nutrition Examination Survey spanning 2005–2018, involving 35,365 participants after applying exclusion criteria. The primary exposure variable was AIP, categorized into quartiles, while the primary outcome variable was CRS, defined by the coexistence of cardiovascular disease (CVD) and chronic kidney disease (CKD). Statistical analyses, considering sample weights, included ANOVA, Chi-square tests, logistic regression models, and restricted cubic spline (RCS) analysis to examine nonlinear relationships.

**Results:**

The weighted logistic regression analysis showed a positive correlation between AIP and CRS across all models. In the fully adjusted model, the highest AIP quartile had a significantly increased odds ratio (OR) for CRS (Q4: OR = 1.62; 95% CI: 1.21–2.15). RCS analysis confirmed a positive correlation between AIP and CRS, with TG positively and HDL-C negatively correlated with CRS. Subgroup analysis indicated a significant interaction with hypertension, showing a stronger association in non-hypertensive individuals.

**Conclusion:**

Higher AIP levels are associated with an increased prevalence of CRS, with a notable hypertension-specific interaction indicating a higher effect in individuals without hypertension.

## Introduction

Cardiorenal syndrome (CRS) is a complex clinical condition characterized by the simultaneous dysfunction of the heart and kidneys [1]. This bidirectional relationship can manifest in acute or chronic forms, where the failure of one organ can precipitate failure in the other [2–4]. The syndrome is divided into five subtypes based on the nature and timeline of organ dysfunction [2,5,6]. It significantly contributes to morbidity and mortality, particularly in patients with coexisting chronic diseases [7]. Epidemiological data reveal that CRS prevalence is notably high among patients with chronic kidney disease (CKD) and cardiovascular disease (CVD), underscoring the critical need for early diagnosis and targeted interventions [7,8].

The Atherogenic Index of Plasma (AIP) is an established biomarker that has been extensively studied for its potential role in predicting cardiovascular risk [9]. AIP is calculated as the logarithm of the ratio between triglycerides (TG) and high-density lipoprotein cholesterol (HDL-C) [10]. Elevated AIP levels are associated with an increased presence of small, dense low-density lipoprotein (LDL) particles, which are more prone to oxidation and thus more atherogenic [11]. Previous studies have demonstrated that AIP is a reliable predictor of cardiovascular events and can reflect the overall state of atherosclerosis better than traditional lipid measures alone [10,12,13]. On the other hand, dyslipidemia is highly prevalent in the context of renal diseases, particularly in patients with end-stage renal disease. This condition is typically characterized by elevated triglyceride levels and reduced HDL-C levels [14,15]. Studies have demonstrated that patients undergoing hemodialysis exhibit significantly higher AIP levels [16]. Dyslipidemia is recognized as a critical risk factor influencing cardio-renal-metabolic health, underscoring the need for comprehensive management strategies targeting lipid abnormalities in this population [17].

Despite the growing body of evidence linking lipid abnormalities with cardiovascular outcomes [18–20], the relationship between AIP and CRS remains underexplored. Given the intertwined pathophysiology of heart and kidney dysfunction, understanding how AIP correlates with CRS could provide valuable insights for clinical practice. This study aims to fill this gap by investigating the association between AIP and CRS, leveraging a large dataset to determine the possible relationship between AIP and CRS.

## Methods

### Data source and participants selection

The National Health and Nutrition Examination Survey (NHANES) is a critical resource for public health research in the United States, providing comprehensive data on the health and nutritional status of the civilian, non-institutionalized population. Established in the early 1970s, NHANES employs a cross-sectional design to collect data through interviews, physical examinations, and laboratory tests, thereby enabling a multifaceted assessment of health indicators across diverse demographic groups [21]. The survey’s data collection methodology is rigorous, involving trained personnel who ensure the accuracy and reliability of self-reported dietary information [22].

**Figure 1.**
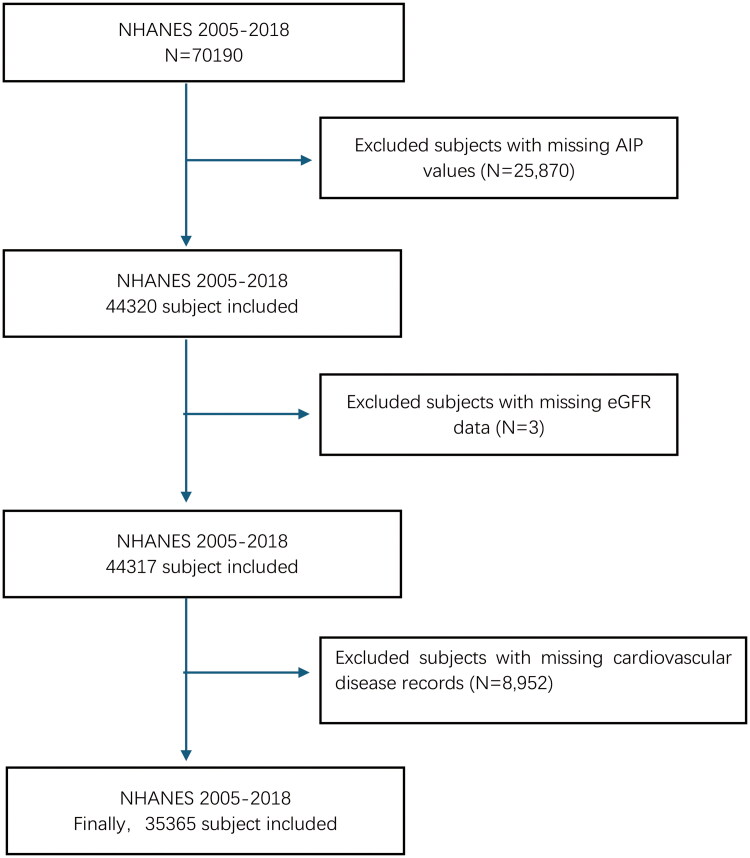
Flow chart of participants selection.

In this study, data from seven NHANES survey cycles (2005–2018) were included, encompassing a total of 70,190 participants. All participants provided written informed consent, and the study protocols were approved by the NCHS Ethics Review Board [23]. Participants were excluded if they lacked complete information on TG and HDL-C, resulting in the exclusion of 25,870 individuals. Additionally, those missing data on serum creatinine (*n* = 3) and those without questionnaire data on congestive heart failure, coronary heart disease, angina pectoris, heart attack, and stroke (*n* = 8,952) were also excluded from the analysis (Figure 1). After applying these exclusion criteria, a final sample of 35,365 participants was included in the study.

### Exposure variable and outcome variables

The primary exposure variable in this study was the AIP, which was calculated using the formula AIP = log10(TG(mmol/L)/HDL-C(mmol/L)) [24]. Based on their AIP values, participants were categorized into four quartiles. Additional secondary exposure variables included TG (mmol/L), total cholesterol (TC, mmol/L), and HDL-C(mmol/L).

The outcome variable was CRS, defined as the simultaneous presence of CVD and CKD in an individual [18,25], as referenced in prior NHANES studies. CVD was identified through self-reported diagnoses, including coronary heart disease, angina pectoris, congestive heart failure, heart attack, or stroke [25]. CKD was determined by estimating the glomerular filtration rate (eGFR), with eGFR values below 60 mL/min per 1.73 m^2^ indicating CKD. The CKD-Epidemiology Collaboration (CKD-EPI) equation was used to calculate eGFR:

$$\begin{array}{l} \text{eGFR}=141\times \text{min}{\left(\text{Scr}/k,1\right)}^{\alpha }\times \text{max}{\left(\text{Scr}/k,1\right)}^{-1.209}\times 0.993^{\text{age}}\\ \;\times 1.018\left[\text{iffemale}\right]\times 1.159\left[\text{ifblack}\right], \end{array}$$

Where Scr denotes serum creatinine concentration (mg/dL) measured by the Jaffe rate method. The constant *k* is 0.9 for males and 0.7 for females, and the exponent *α* is −0.411 for males and −0.329 for females [26].

### Covariates

The study incorporated several covariates to control for potential confounding factors, selected based on their relevance to the exposure and outcome variables and included: Age, Gender, Race, Education, Marital Status, Ratio of household income to poverty (PIR), Alcohol, Smoking Status, Diabetes, Hypertension (Supplemental Table S1).

### Statistical analysis

In this study, missing data were addressed using multiple imputation methods [27], and appropriate weighting techniques were employed according to NHANES guidelines to account for the complex sampling design, ensuring nationally representative results. The exposure variable, AIP and the other three exposure variables, was divided into four quartiles with the lowest quartile (Q1) serving as the reference group. Analysis of variance (ANOVA) was used to compare quantitative data between groups, while Chi-square tests or Fisher’s exact tests were used for qualitative data comparisons. Multivariate logistic regression models were constructed to explore the relationship between AIP and CRS, with three models: Model 1 was unadjusted, Model 2 adjusted for age, gender, and race, and Model 3 (fully adjusted model) included adjustments for age, gender, race, education, marital status, household income, smoking status, alcohol consumption, hypertension, and diabetes. Additionally, restricted cubic spline (RCS) analysis was performed to determine the nonlinear relationship between AIP and CRS, and subgroup analyses were conducted to evaluate whether the relationship between AIP and CRS differed significantly across various subgroups. All statistical analyses were conducted using R version 4.3.2, with a *p*-value of less than 0.05 considered statistically significant.

## Results

### Baseline characteristics of the study population

The study included a total of 35,365 participants, among whom 1162 individuals were diagnosed with CRS. Participants were divided into four groups based on the quartiles of their AIP (Table 1). Higher AIP quartiles were associated with older age groups (Q1 to Q4: 45.21 ± 17.28, 47.07 ± 17.43, 48.68 ± 17.03, 48.65 ± 15.49). The proportion of male participants also increased across the quartiles, from 34.17% in Q1 to 64.87% in Q4.

**Table 1.** Weighted baseline characteristics of the population grouped by AIP quartiles.

Characteristic	Overall, *N* = 35,365 (100%)	Q1 (−1.3,−0.22)	Q2 (−0.22,0.01)	Q3 (0.01,0.26)	Q4 (0.26,1.81)	*p* Value
Age	47.37 ± 16.90	45.21 ± 17.28	47.07 ± 17.43	48.68 ± 17.03	48.65 ± 15.49	<0.001
TC	5.04 ± 1.08	4.83 ± 0.97	4.93 ± 1.00	5.05 ± 1.09	5.35 ± 1.19	<0.001
TG	1.73 ± 1.45	0.74 ± 0.21	1.17 ± 0.27	1.71 ± 0.39	3.40 ± 2.06	<0.001
HDL-C	1.39 ± 0.43	1.80 ± 0.43	1.46 ± 0.30	1.25 ± 0.25	1.01 ± 0.22	<0.001
Gender						<0.001
Male	17,110 (48.20%)	3,263 (34.17%)	3,867 (43.76%)	4,483 (51.01%)	5,497 (64.87%)	
Female	18,255 (51.80%)	5,731 (65.83%)	4,839 (56.24%)	4,457 (48.99%)	3,228 (35.13%)	
Race						<0.001
Non-Hispanic White	14,992 (67.39%)	3,522 (65.51%)	3,620 (67.37%)	3,913 (68.02%)	3,937 (68.79%)	
Non-Hispanic Black	7,372 (10.78%)	2,877 (16.76%)	2,057 (11.99%)	1,507 (8.53%)	931 (5.45%)	
Mexican American	5,617 (8.56%)	871 (5.60%)	1,293 (8.00%)	1,582 (9.53%)	1,871 (11.31%)	
Other Hispanic	3,407 (5.57%)	705 (4.78%)	790 (5.35%)	944 (5.88%)	968 (6.33%)	
Other Race	3,977 (7.69%)	1,019 (7.35%)	946 (7.29%)	994 (8.04%)	1,018 (8.12%)	
Education						<0.001
Less than high school	8,807 (16.03%)	1,728 (12.19%)	2,071 (15.18%)	2,374 (17.24%)	2,634 (19.75%)	
High school graduate or GED	8,074 (23.12%)	1,893 (20.06%)	2,018 (22.84%)	2,091 (24.42%)	2,072 (25.34%)	
University or above	18,484 (60.85%)	5,373 (67.74%)	4,617 (61.98%)	4,475 (58.33%)	4,019 (54.92%)	
Marital status						<0.001
Married/Living with a partner	21,249 (63.92%)	4,828 (59.90%)	5,060 (61.57%)	5,578 (65.85%)	5,783 (68.65%)	
Widowed/Divorce/Separated	7,792 (18.36%)	1,971 (17.87%)	2,009 (18.94%)	1,993 (18.45%)	1,819 (18.19%)	
Never married	6,324 (17.72%)	2,195 (22.23%)	1,637 (19.49%)	1,369 (15.70%)	1,123 (13.15%)	
PIR						<0.001
<1.3	11,323 (21.63%)	2,608 (19.63%)	2,692 (20.60%)	2,911 (22.49%)	3,112 (23.95%)	
1.3–3.5	13,402 (35.84%)	3,346 (34.05%)	3,363 (36.75%)	3,394 (35.94%)	3,299 (36.71%)	
>3.5	10,640 (42.53%)	3,040 (46.32%)	2,651 (42.65%)	2,635 (41.57%)	2,314 (39.34%)	
Smoking status						<0.001
Never	19,641 (55.20%)	5,538 (60.88%)	4,994 (57.56%)	4,801 (53.05%)	4,308 (48.94%)	
Smoking cessation	8,512 (24.65%)	1,852 (21.94%)	2,004 (23.23%)	2,302 (26.24%)	2,354 (27.38%)	
Smoking at present	7,212 (20.14%)	1,604 (17.18%)	1,708 (19.21%)	1,837 (20.71%)	2,063 (23.69%)	
Alcohol						<0.001
Yes	23,922 (72.92%)	6,193 (75.00%)	5,834 (71.88%)	5,907 (71.78%)	5,988 (72.92%)	
No	11,443 (27.08%)	2,801 (25%)	2,872 (28.12%)	3,033 (28.22)	2,737 (27.08%)	
Hypertension						<0.001
Yes	15,138 (38.04%)	3,030 (27.52%)	3,551 (35.01%)	4,187 (42.37%)	4,370 (47.94%)	
No	20,227 (61.96%)	5,964 (72.48%)	5,155 (64.99%)	4,753 (57.63%)	4,355 (52.06%)	
Diabetes						<0.001
Yes	6,223 (13.11%)	807 (5.99%)	1,251 (9.84%)	1,861 (15.61%)	2,304 (21.50%)	
No	29,142 (86.89%)	8,187 (94.01%)	7,455 (90.16%)	7,079 (84.39%)	6,421 (78.5%)	
CRS						<0.001
Without CRS	34,203 (97.63%)	8,805 (97.9%)	8,452 (97.1%)	8,579 (96%)	8,367 (95.9%)	
With CRS	1,162 (2.37%)	189 (2.1%)	254 (2.9%)	361 (4%)	358 (4.1%)	

Mean ± standard deviation for continuous variables; *N* (%) for categorical variables.

Significant differences were observed across the quartiles for other demographic and clinical characteristics, including race, education level, marital status, household income, smoking status, alcohol consumption, hypertension, and diabetes (*p* < 0.05). Notably, participants with higher AIP levels had a greater prevalence of CRS. Specifically, the prevalence of CRS was 4% in Q3 and 4.1% in Q4, compared to 2.1% in Q1 and 2.9% in Q2. Additionally, levels of TC, TG, and HDL-C also showed significant differences (*p* < 0.05).

### Logistic regression analysis

To investigate the association between the AIP and CRS, we constructed three logistic regression models. When AIP was treated as a continuous variable, all three models demonstrated a positive correlation between AIP and CRS (Table 2). Similarly, when AIP was categorized into quartiles, a significant association between AIP and CRS was observed across all models, and an increasing effect size with higher AIP values.

**Table 2.** The weighted logistic regression relationship between AIP and CRS.

	Model 1		Model 2		Model 3	
	OR (95%CI)	P	OR (95%CI)	P	OR (95%CI)	P
AIP (continuous)	2.07 (1.66,2.59)	*p* < 0.001	3.01 (2.24,4.04)	*p* < 0.001	1.91 (1.43,2.56)	*p* < 0.001
Q1	Reference		Reference		Reference	
Q2	1.30 (1.01,1.67)	*p* = 0.04	1.18 (0.90,1.55)	*p* = 0.2	1.06 (0.82,1.37)	*p* = 0.7
Q3	1.84 (1.42,2.40)	*p* < 0.001	1.66 (1.24,2.23)	*p* < 0.001	1.33 (0.99,1.78)	*p* = 0.06
Q4	2.04 (1.57,2.67)	*p* < 0.001	2.36 (1.77,3.16)	*p* < 0.001	1.62 (1.21,2.15)	*p* = 0.001

Model 1 was the crude model; Model 2 was adjusted for age, gender, and race; Model 3 was adjusted for age, gender, race, education level, marital status, PIR, alcohol consumption, smoking status, diabetes mellitus, and hypertension.

In Model 3, although Q2 and Q3 did not show a statistically significant difference compared to Q1, the odds ratios (OR) increased markedly from Q2 to Q4. The specific OR values were as follows: Q2: OR = 1.06 (95% CI: 0.82–1.37; *p* = 0.7), Q3: OR = 1.33 (95% CI: 0.99–1.78; *p* = 0.06), Q4: OR = 1.62 (95% CI: 1.21–2.15; *p* = 0.001)

Additionally, we constructed regression models using the quartiles of TC, TG, and HDL-C as grouping variables. The relationships between TC, TG, and HDL-C with CRS were inverse, direct, and inverse, respectively (Supplemental Table S2).

### Nonlinear relationships

**Figure 2.**
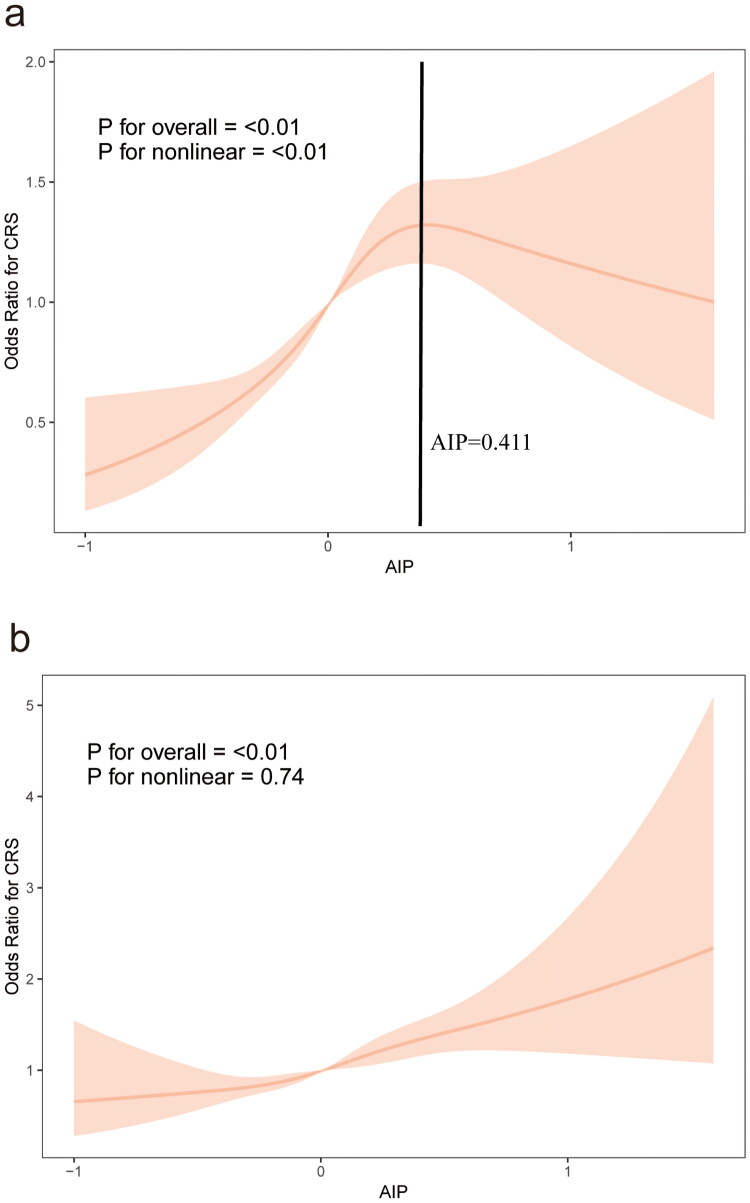
A weighted restricted cubic spline plot of AIP and CRS with 4 knots was generated. (a) No adjustment was made for covariates. (b) Adjustments were made for age, gender, race, education level, marital status, PIR, alcohol consumption, smoking status, diabetes mellitus, and hypertension. The solid line represents the estimated values, and the shaded area in the figure represents the 95% confidence interval of the or.

To further validate our findings, we used RCS analysis to examine the nonlinear relationship between AIP and CRS. As shown in Figure 2, Figure 2(a) illustrates the relationship between AIP and CRS without adjusting for covariates, showing a positive correlation when AIP values are below 0.411 and a negative correlation when AIP values exceed this threshold (P for nonlinear < 0.01). In contrast, Figure 2(b) demonstrates a consistent positive correlation between AIP and CRS in the fully adjusted model (P for nonlinear = 0.74).

**Figure 3.**
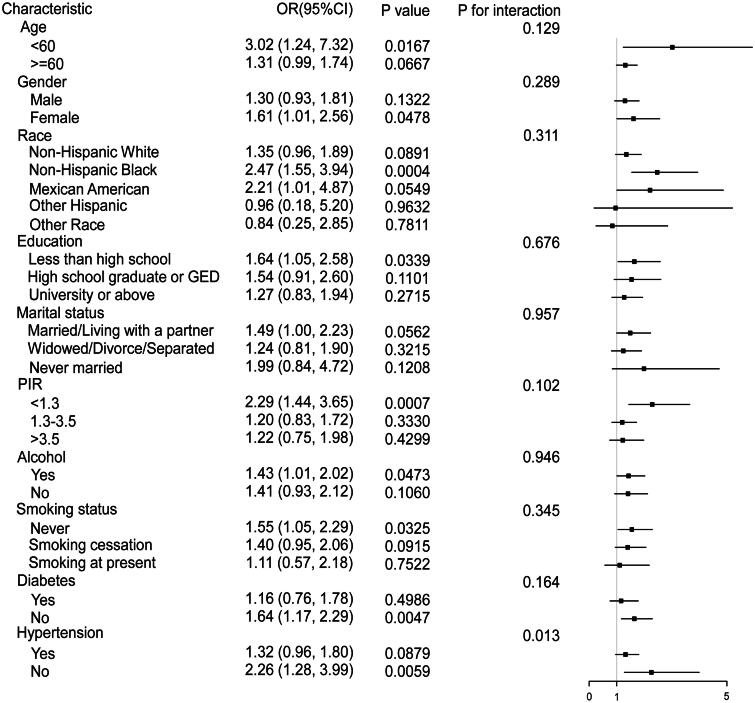
Weighted subgroup analysis of the association between AIP and CRS. The model adjusted for age, gender, race, education level, marital status, PIR, alcohol consumption, smoking status, diabetes mellitus, and hypertension in all subgroups, with the exception of the stratification variable.

Similarly, TG showed a positive correlation with CRS, and HDL-C exhibited a negative correlation with CRS (Supplemental Figures S1 and S2). The relationship between TC and CRS was more complex: TC was negatively correlated with CRS when cholesterol levels were below 5.867 mmol/L, but positively correlated with CRS at higher concentrations (Supplemental Figure S3).

### Subgroup analysis and interaction test

Subgroup analyses and interaction tests were conducted to examine whether the relationship between the AIP and CRS was consistent across different demographic and clinical subgroups. As shown in Figure 3, participants were stratified by age, gender, race, education level, marital status, household income, smoking status, alcohol and diabetes. The analyses indicated that the association between AIP and CRS did not differ significantly across these subgroups (P for interaction > 0.05). However, a significant interaction was identified in the stratified analysis by hypertension status (P for interaction = 0.013), indicating that the association between AIP and CRS differs between individuals with and without hypertension.

## Discussion

This study utilized data from a large population-based cohort of the U.S. population to investigate the relationship between the AIP and CRS. Using weighted logistic regression, our findings indicate that higher AIP levels are associated with an increased prevalence of CRS among Americans. Moreover, this relationship exhibits a significant difference between hypertension, suggesting that the impact of AIP on CRS risk may vary between individuals with and without hypertension.

Notably, the prevalence of CRS escalated with higher AIP quartiles, a trend consistent with the findings of Ensling et al., [19] who identified that insulin resistance and metabolic dyslipidemia, characterized by elevated non-esterified fatty acids (NEFA) and TG, contribute significantly to the development of CRS. Furthermore, employing RCS analysis, our study elucidated the non-linear relationships between AIP and CRS, where TG demonstrated a positive correlation with CRS, whereas HDL-C exhibited a negative correlation. These intricate relationships are corroborated by a study conducted in Saudi Arabia [20], which found that various lipid parameters, such as TG and HDL-C, had differential impacts on cardiovascular risk across distinct patient subgroups.

The positive correlation between TG and CRS observed in our study may be attributed to TG’s role in promoting atherosclerosis and endothelial dysfunction, which are critical factors in cardiovascular and renal impairments [28–30]. Elevated TG levels lead to increased production of atherogenic lipoproteins, resulting in systemic inflammation and oxidative stress, which in turn exacerbate cardiac and renal dysfunction [1,31]. Conversely, HDL-C is known for its protective cardiovascular effects, including reverse cholesterol transport, anti-inflammatory properties, and endothelial protection [3,32,33]. Lower HDL-C levels are associated with a higher risk of atherosclerosis and subsequent cardiovascular events [34,35]. The negative correlation between HDL-C and CRS observed in our study aligns with these protective roles of HDL-C.

AIP, as an indicator of dyslipidemia, reflects the balance between TG and HDL-C, with elevated AIP levels indicating a higher TG/HDL-C ratio. In the unadjusted Model 1, a nonlinear relationship between AIP and CRS was observed, possibly due to the influence of confounding factors. However, in the fully adjusted Model 3, a positive association between AIP levels and CRS emerged. This finding aligns with the concept that elevated TG levels and reduced HDL-C concentrations exacerbate the risk and progression of CRS, underscoring the critical role of atherogenic dyslipidemia in the development of cardio-renal dysfunction [17,20]. Notably, in multivariate regression Model 3, the difference between Q1 and Q2 was not statistically significant, likely due to both groups being in the low-risk category for AIP values [36,37].

Previous studies have consistently demonstrated that hypertension and diabetes are significantly associated with the presence or absence of CRS [38–40]. In the subgroup analysis, our study found a significant difference in the association between AIP and CRS across hypertensive and non-hypertensive populations. Chronic hypertension damages the vascular endothelium, increasing the risk of endothelial dysfunction and atherosclerosis, which can lead to cardiovascular events and renal impairment [41,42]. Additionally, prolonged hypertension induces structural and functional changes in the heart, such as left ventricular hypertrophy (LVH), which heightens the risk of heart failure and arrhythmias [43,44].

The presence of hypertension in patients with CKD is associated with accelerated disease progression, increased proteinuria, and a heightened risk of cardiovascular complications [42,43]. Studies have demonstrated that effective management of hypertension can slow CKD progression and improve cardiovascular outcomes [45]. Therefore, in hypertensive individuals, preexisting cardiovascular and renal damage caused by elevated blood pressure may have masked the effect of AIP on CRS (OR = 1.32; 95% CI: 0.96–1.8). In contrast, in non-hypertensive individuals, the effect of AIP on CRS was more pronounced, with an increase in effect size of 94% (OR = 2.26; 95% CI: 1.28–3.99). A similar pattern was observed in the diabetes subgroup analysis, where the effect of AIP on CRS was 48% greater in non-diabetic individuals compared to those with diabetes.

This study possesses several notable strengths. Firstly, the large sample size confers substantial statistical power, enabling the detection of significant associations. Secondly, the employment of multivariate regression models facilitated the adjustment for multiple confounding factors, thereby enhancing the validity of the findings. Thirdly, conducting subgroup analyses allowed for the assessment of the consistency of the AIP-CRS association across various demographic and clinical subgroups, thereby augmenting the generalizability of the results. Our analysis showed no statistically significant differences across subgroups, except for the hypertension subgroup, suggesting that the study results may be applicable to populations with diverse demographic characteristics, including different ethnicities, age groups, and educational backgrounds.

However, this study is not without limitations. As a cross-sectional design, it inherently cannot establish causality between AIP and CRS. In patients with CKD, lipid metabolism disorders may be attributed to several factors, including impaired tubular function, metabolic syndrome, and endocrine disturbances [46–48]. These complex interactions may confound the observed associations and warrant further longitudinal studies to clarify the causal pathways linking AIP and CRS. Despite the adjustment for numerous confounders, the possibility of residual confounding cannot be entirely excluded. Furthermore, the data for CVD and other comorbidities were self-reported, which may introduce misclassification bias. Our study sample was derived through complex multistage sampling, taking into account the sample weights, which allows for a reflection of the broader U.S. population. However, the findings from this study still require further validation and replication through additional studies to ensure their generalizability and applicability.

## Conclusion

Higher AIP levels are associated with an increased prevalence of CRS, with a notable hypertension-specific interaction indicating a higher effect in individuals without hypertension.

## Supplementary Material

Additional file 1.docx

## Data Availability

The dataset for this study is accessible through a publicly available repository, please visit the website at: https://www.cdc.gov/nchs/nhanes/.
