# Supplementary material for: Elevated atherogenic index of plasma is associated with increased cardiorenal syndrome prevalence: a cross-sectional study
Source: Ren Fail. 2025 Mar 2;47(1):2472037. doi: 10.1080/0886022X.2025.2472037 (PMC11878164; doi:10.1080/0886022X.2025.2472037)
Supplement: Additional file 1.docx [file IRNF_A_2472037_SM8053.docx]

**Supporting information**

**Fig S1** A weighted restricted cubic spline plot of TG and CRS with 4 knots was generated. Adjustments were made for age, gender, race, education level, marital status, PIR, alcohol consumption, smoking status, diabetes mellitus, and hypertension. The solid line represents the estimated values, and the shaded area in the figure represents the 95% confidence interval of the OR.

**Fig S2** A weighted restricted cubic spline plot of HDL and CRS with 4 knots was generated. Adjustments were made for age, gender, race, education level, marital status, PIR, alcohol consumption, smoking status, diabetes mellitus, and hypertension. The solid line represents the estimated values, and the shaded area in the figure represents the 95% confidence interval of the OR.

**Fig S3** A weighted restricted cubic spline plot of TC and CRS with 4 knots was generated. Adjustments were made for age, gender, race, education level, marital status, PIR, alcohol consumption, smoking status, diabetes mellitus, and hypertension. The solid line represents the estimated values, and the shaded area in the figure represents the 95% confidence interval of the OR.

**Table S1** **Covariates and Definitions**

| **Variable Name** | **Definition** |
| --- | --- |
| Age(years) | Continuous variable |
| Sex | Male or female |
| Race | Non-Hispanic white, non-Hispanic black, Mexican American, other Hispanic, or other |
| Education | Less than high school, high school graduate, and more than high school |
| Marital Status | Married/Living with a partner, Widowed/Divorce/Separated, Never married |
| PIR | Less than 1.30, 1.31 to 3.50, and greater than 3.50 |
| Alcohol | Participants consuming alcohol more than 12 times in the past year classified as drinkers; otherwise categorized as non-drinkers |
| Smoking Habits | never smokers (smoked fewer than 100 cigarettes in their lifetime), former smokers (smoked more than 100 cigarettes but are not currently smoking), and current smokers (smoked more than 100 cigarettes and are currently smoking) |
| Diabetes | Based on history of diabetes, use of insulin or oral hypoglycemic agents, HbA1c levels of 6.5% or higher, or fasting glucose levels of 126 mg/dL or higher |
| Hypertension | SBP of 140 mmHg or higher, DBP of 90 mmHg or higher, or self-reported hypertension with use of antihypertensive medication |

**Table S2** **The weighted logistic regression relationship of TG, HDL, and TC with CRS**

|  | **Model1** | | **Model2** | | **Model3** | |
| --- | --- | --- | --- | --- | --- | --- |
|  | OR (95%CI) | P | OR (95%CI) | P | OR (95%CI) | P |
| **TC**  **(continuous)** | 0.61  (0.55, 0.67) | p<0.001 | 0.65  (0.59, 0.72) | p<0.001 | 0.73  (0.66, 0.80) | p<0.001 |
| **Q1**  **(0.16，4.24）** | Reference | | Reference | | Reference | |
| **Q2**  **(4.24，4.91)** | 0.40  (0.34, 0.49) | p<0.001 | 0.48  (0.39, 0.59) | p<0.001 | 0.56  (0.45, 0.69) | p<0.001 |
| **Q3**  **(4.91，5.66）** | 0.28  (0.23, 0.35) | p<0.001 | 0.31  (0.25, 0.39) | p<0.001 | 0.41  (0.32, 0.52) | P<0.001 |
| **Q4**  **(5.66，16.78)** | 0.34  (0.28, 0.41) | p<0.001 | 0.34  (0.27, 0.42) | p<0.001 | 0.45  (0.35, 0.56) | p<0.001 |
| **TG**  **(continuous)** | 1.06  (1.03, 1.09) | p<0.001 | 1.12  (1.08, 1.18) | p<0.001 | 1.08  (1.03, 1.13) | p=0.004 |
| **Q1**  **(-2.28，-0.09)** | Reference | | Reference | | Reference | |
| **Q2**  **(-0.09，0.31)** | 1.86  (1.39, 2.48) | p<0.001 | 1.41  (1.03, 1.93) | p=0.032 | 1.29  (0.94, 1.77) | p=0.11 |
| **Q3**  **(0.31，0.75)** | 2.24  (1.69, 2.96) | p<0.001 | 1.65  (1.22, 2.23) | P=0.001 | 1.37  (1.00, 1.87) | p=0.048 |
| **Q4**  **(0.75，4.23)** | 2.37  (1.78, 3.17) | p<0.001 | 2.11  (1.55, 2.88) | p<0.001 | 1.52  (1.11, 2.09) | p=0.01 |
| **HDL**  **(continuous)** | 0.61  (0.49, 0.75) | p<0.001 | 0.33  (0.25, 0.43) | p<0.001 | 0.48  (0.37, 0.62) | p<0.001 |
| **Q1**  **(0.16，1.09)** | Reference | | Reference | | Reference | |
| **Q2**  **(1.09，1.32)** | 0.83  (0.68, 1.00) | p=0.056 | 0.69  (0.56, 0.85) | p<0.001 | 0.78  (0.63, 0.96) | p=0.019 |
| **Q3**  **(1.32，1.60)** | 0.64  (0.52, 0.78) | p<0.001 | 0.44  (0.36, 0.54) | p<0.001 | 0.56  (0.45, 0.70) | p<0.001 |
| **Q4**  **(1.60，5.84)** | 0.59  (0.49, 0.72) | p<0.001 | 0.32  (0.26, 0.40) | p<0.001 | 0.46  (0.37, 0.59) | p<0.001 |

Model 1 was the crude model; Model 2 was adjusted for age, gender, and race; Model 3 was adjusted for age, gender, race, education level, marital status, PIR, alcohol consumption, smoking status, diabetes mellitus, and hypertension.
